# Flood Control: How Milk-Derived Extracellular Vesicles Can Help to Improve the Intestinal Barrier Function and Break the Gut–Joint Axis in Rheumatoid Arthritis

**DOI:** 10.3389/fimmu.2021.703277

**Published:** 2021-07-28

**Authors:** Joyce Aarts, Annemarie Boleij, Bartijn C. H. Pieters, Anouk L. Feitsma, R. J. Joost van Neerven, Jean Paul ten Klooster, Laura M’Rabet, Onno J. Arntz, Marije I. Koenders, Fons A. J. van de Loo

**Affiliations:** ^1^Department of Rheumatology, Radboud Institute for Molecular Life Sciences, Radboud University Medical Center (Radboudumc), Nijmegen, Netherlands; ^2^Department of Pathology, Radboud Institute for Molecular Life Sciences, Radboud University Medical Center (Radboudumc), Nijmegen, Netherlands; ^3^FrieslandCampina, Amersfoort, Netherlands; ^4^Cell Biology and Immunology, Wageningen University & Research, Wageningen, Netherlands; ^5^Research Centre for Healthy and Sustainable Living, Innovative Testing in Life Sciences and Chemistry, University of Applied Sciences, Utrecht, Netherlands

**Keywords:** rheumatoid arthritis, intestine, microbiome, immunomodulation, extracellular vesicles, bovine milk

## Abstract

Many studies provided compelling evidence that extracellular vesicles (EVs) are involved in the regulation of the immune response, acting as both enhancers and dampeners of the immune system, depending on the source and type of vesicle. Research, including ours, has shown anti-inflammatory effects of milk-derived EVs, using human breast milk as well as bovine colostrum and store-bought pasteurized cow milk, in *in vitro* systems as well as therapeutically in animal models. Although it is not completely elucidated which proteins and miRNAs within the milk-derived EVs contribute to these immunosuppressive capacities, one proposed mechanism of action of the EVs is *via* the modulation of the crosstalk between the (intestinal) microbiome and their host health. There is increasing awareness that the gut plays an important role in many inflammatory diseases. Enhanced intestinal leakiness, dysbiosis of the gut microbiome, and bowel inflammation are not only associated with intestinal diseases like colitis and Crohn’s disease, but also characteristic for systemic inflammatory diseases such as lupus, multiple sclerosis, and rheumatoid arthritis (RA). Strategies to target the gut, and especially its microbiome, are under investigation and hold a promise as a therapeutic intervention for these diseases. The use of milk-derived EVs, either as stand-alone drug or as a drug carrier, is often suggested in recent years. Several research groups have studied the tolerance and safety of using milk-derived EVs in animal models. Due to its composition, milk-derived EVs are highly biocompatible and have limited immunogenicity even cross species. Furthermore, it has been demonstrated that milk-derived EVs, when taken up in the gastro-intestinal tract, stay intact after absorption, indicating excellent stability. These characteristics make milk-derived EVs very suitable as drug carriers, but also by themselves, these EVs already have a substantial immunoregulatory function, and even without loading, these vesicles can act as therapeutics. In this review, we will address the immunomodulating capacity of milk-derived EVs and discuss their potential as therapy for RA patients.

**Review criteria:**

The search terms “extracellular vesicles”, “exosomes”, “microvesicles”, “rheumatoid arthritis”, “gut-joint axis”, “milk”, and “experimental arthritis” were used. English-language full text papers (published between 1980 and 2021) were identified from PubMed and Google Scholar databases. The reference list for each paper was further searched to identify additional relevant articles.

## Introduction

There is increasing awareness that the gut plays an important role in many inflammatory diseases. The intestinal epithelial cell layer is a selectively permeable barrier permitting the absorption of nutrients, but at the same time preventing the entry of microorganisms (gut flora/microbiome) (**Box 1**). The gut also has an active immune surveillance system to actually cope with these microbes and is the largest immune organ of the body (11). Enhanced gut leakiness, dysbiosis, and intestinal inflammation are associated with the pathogenesis of many inflammatory and autoimmune diseases, such as Crohn’s disease and rheumatoid arthritis (RA) (12–14). Patients with these diseases also frequently report enhanced disease activity after food intake (15). The relationship between food intake and enhanced disease activity is further supported by antibodies against food components in the blood of these patients (15).

Box 1 **Gut microbiome and gastro-intestinal function.**

The gut microbiome consists of bacteria, bacteriophages, yeasts, protozoa, and viruses and can be seen as an external organ. The biggest component of the gut microbiome are bacteria. Colonization with gut microbes starts after birth and depends on many external factors, such as the delivery mode, type of feeding (breast versus formula), maternal factors, and other early life exposures such as infections or use of antibiotics (1, 2). The gut microbiota in early life is important for the maturation of the immune system, and it produces vitamins, minerals, and energy from our diet (3). During childhood, a complex relation between the host and its microbiome develops that stabilizes over time (4, 5). The developed host–microbiome symbiosis is essential for health throughout life. After coevolution of the immune system with the microbiome, keeping the balance is of utmost importance to sustain health. Microbiome disruptions can therefore lead to changes in barrier function and immune responses that contribute to disease development or progression (6). In this respect, a highly diverse microbiome is considered healthy, as it helps to free essential nutrients and energy, helps detoxification of toxic substances such as primary bile acids, and provides colonization resistance against pathogens. Contrarily, a low diversity is linked to microbial dysbiosis and associated with many diseases, including autoimmune disorders such as RA (7, 8). However, there is still debate whether higher diversity is always a good thing (9). Keeping a balanced microbiome is therefore essential for the function of our gut and maintaining health. A diverse diet rich in fibers, polyphenols, and fermented food helps to maintain a healthy microbiome that provides short-chain fatty acids and essential vitamins that are important energy sources for the gut epithelium (10). It is clear that a delicate balance between the host and its microbiome exists that reflects our health and is influenced by many external factors of which lifestyle is the most important.

The pathogenesis of most autoimmune diseases is poorly understood, but environmental factors, including the microbiome, and genetic background are known to play a role in the development of these disorders (16). Autoimmunity is breaking self-tolerance and one of the proposed mechanisms is epitope mimicry, a cross reactive immune recognition of self and viral or bacterial epitopes (17). Some bacteria are capable of post-translational modification of body’s own proteins by citrullination creating altered self-epitopes (18). Citrullination is catalyzed by host’s own but also bacterial peptidylarginine deiminase (PAD) enzymes (18). There is compelling preclinical evidence that the gut microbiome is causally related to this break in self-tolerance and clinically a leaky gut is linked with a higher risk of autoimmune diseases (12). The microbiome consists of all living microorganisms of a defined region, such as the gastro-intestinal tract. Multiple lines of evidence support the potential pathogenic role of microbial gut dysbiosis in inflammatory disorders of the intestine, but also in autoimmune disorders such as RA, indicating an important role for the gut–joint axis in the development of this disease (19). For instance, in experimental arthritis, RA disease is strongly attenuated in germ-free (GF) mice compared to conventionally colonized mice, as was also reported for experimental autoimmune encephalomyelitis (20, 21). Both systemic and intestinal T-helper 17 (Th17) cell differentiation was strongly reduced in these GF mice (20–22), indicating an important role of the microbiome in breaking immune tolerance. Also, targeting intestinal barrier dysfunction before arthritis onset attenuates development of collagen-induced arthritis (23). This makes the gut and its microbiota promising targets for drug- and dietary intervention (24). A way of doing this is to optimize the micromilieu for hosting favorable microorganisms and at the same time increase the barrier function and direct the immune surveillance to target the putative pathogens and prevent their entry. In this sense, antibiotics are like a sledgehammer, and although promising results are obtained in animal models (25, 26), the use of antibiotics is also linked to microbiome dysbiosis and consequently the development of autoimmune disease. Probiotics and prebiotics to modulate the microbiome and thereby the gut–joint axis are currently under investigation (27); also, immune-regulatory components from food are promising options. Milk is a complex biological fluid with unique bioactive components that influence gut immunity, intestinal flora, and growth and development of infants (28, 29). Breastfeeding is associated with a decreased risk of asthma and allergic disease during childhood [reviewed in (30)]. However, a protective effect of breastfeeding against atopy, eczema, and food allergies is not convincingly proven yet (30, 31). On the other hand, several studies indicate a protective effect of raw cow milk consumption early in life against the development of asthma and respiratory tract infections during childhood (32–37). However, in some studies, the effects were not always independent of other farm-related exposures, *e.g.* exposure to straw, silage, or cows (32, 33, 36). The underlying mechanisms for this protection are therefore not always clear, but a potential contributor could be extracellular vesicles found in milk. Many proteins present in milk, such as lactoferrin, lactadherin, and immunoglobulins, are implied in mediating these effects.

Compared to milk protein, fat, and hormones, milk-derived exosomes or extracellular vesicles (mEVs) are less frequently studied components of milk (**Box 2**). Our lab has been on the forefront of researching the functional effects of milk EVs on bone and joint-related diseases. Our initial study revealed that milk-derived EVs could attenuate experimental arthritis in mice (14). Oral gavage with milk EVs, or milk EVs in the drinking water of mice resulted in reduced severity of experimental arthritis in two different animal models (14). IL1rn−/− mice developed spontaneous arthritis associated with loss of intestinal microbial diversity and specific taxonomic alterations in the microbiota (50). Furthermore, arthritis in these IL1rn−/− mice was diminished under germ-free conditions and was shown to be dependent on the activation of toll-like receptor 4 (TLR4) and subsequent enhanced Th17 differentiation (22). Interestingly, these mice showed reduced cartilage proteoglycan depletion and bone marrow cellularity after treatment with mEVs by oral gavage. Similarly, in a collagen-induced model for arthritis, where one week before immunization with collagen the mice received milk EVs *via* drinking water, the mEV-treated group showed less severe arthritis. This was accompanied by reduced inflammatory markers in the serum (MCP-1 and IL-6), as well as lower Tbet (Th1) and RORyT (Th17) expression in splenocytes, suggesting reduced T cell activation (14).

Box 2 **Milk processing and milk EV characteristics.**

Bovine milk is part of the human diet. Next to the main milk proteins, *i.e.* caseins and whey proteins, milk contains 3.5% fat present in the milk fat globules, and milk EVs as one of the minor milk components. The structure of milk EVs differs from milk fat globules in the fact that they are membrane vesicles that are structured in a bilayered cell membrane, while the fat globules are surrounded by a trilayered membrane. Milk EVs can be characterized by their size, density, and surface markers like flotillin 1 and tetraspanins CD9 and CD81 (38).

Milk EVs can survive digestion (39, 40), allowing the functional transfer of the bovine milk EVs (including membrane components or EV content) into the human body after consumption (41, 42). However, because raw milk is not sterile and may contain pathogens, processing of milk by heat treatment is required to make bovine milk safe for human consumption. There are several heating methods, from which pasteurization and ultra-heat treatment (UHT) are the processes that are applied most frequently. These processing steps can impact the biological activity of the milk EVs. Pasteurization conditions result in preservation of the milk EVs to a large extent, while UHT is detrimental for the milk EVs and its miRNA (43–46). During milk processing, homogenization is also performed to stabilize the milk fat globules in a uniform way in the milk by decreasing their size. Part of the milk fat globules after homogenization have similar sizes as the milk EVs, and are therefore difficult to differentiate from EVs on the basis of size alone. More pure EVs can be isolated with sucrose gradient centrifugation; however, for the scalability of the milk EVs, this is not the best method (47). To remove protein content and thereby create more pure EVs, acidification is also an option (48, 49).

In this review, we summarize and discuss the current knowledge on the therapeutic potential of bovine milk EVs in inflammatory disorders, in particular in the context of the gut–joint axis in RA.

## Extracellular Vesicles

EVs is the collective term for vesicles secreted by a variety of cells throughout the body and can be found in all body fluids, such as blood, urine, synovial fluid, and milk (51) (**Box 3**). EVs are small cell membrane-derived phospholipid bilayer structures that range in size from 30 to 2,000 nm in diameter (60). Previously, they were considered to be cellular waste products, but compelling evidence has indicated that EVs transport their cargo, consisting of bioactive proteins, enzymes and lipids, and deliver them to recipient cells. This makes EVs important mediators in cell–cell communication.

Box 3 **Biogenesis of EVs.**

Extracellular vesicle is the collective term for vesicles secreted by a variety of cells throughout the body. This heterogeneous population of vesicles is found in body fluids, such as plasma, urine, synovial fluid, milks, saliva, and cerebrospinal fluid (52). A distinction can be made between three different subtypes of vesicles: microvesicles (MVs), apoptotic bodies, and exosomes (53). The nomenclature of these vesicles is still under debate, and ongoing efforts are made to better distinguish vesicle subtypes [see positional paper ISEV (54)]. Within this review, we will use terminology from the original papers. MV size varies from 50 to 1,000 nm (55), making them overlap slightly with exosomes which are 30–150 nm in diameter. Apoptotic bodies are the largest vesicles, ranging from 500 to 2,000 nm. MVs and apoptotic vesicles arise through direct outward budding and fission of the plasma membrane, a process also known as vesicle shedding (56), and by blebbing of the cell membrane during apoptosis (57), respectively. Exosomes, on the other hand, derive from the multivesicular endosome (MVE). The generation of MVEs involves the lateral segregation of cargo at the membrane of an endosome, followed by inward budding and release of vesicles into the endosomal lumen (58). A comprehensive review on the cell biology of EVs was recently published by van Niel et al. (59).

Milk is a rich source of EVs, and EVs obtained from human breast milk as well as from raw and pasteurized cow milk have been characterized in great detail, including their microRNA and protein cargo (48, 61). A large part of highly abundant microRNAs in milk-derived EVs are evolutionary conserved and are present in milk of all mammals (62). Numerous microRNAs have been identified in milk-derived EVs, of which a large number have been described as having an immune-modulatory function. In **Table 1**, a list of these commonly identified microRNAs can be found.

**Table 1:** Commonly identified microRNAs in milk-derived EVs.

MicroRNA present in bovine milk EVs	Expected function
**Let7**	Protection against bacterial infection (63)
**miR-21**	Linked to regulation of TLR signaling (64)
	Clearance of apoptotic cells (65)
	Clearance of bacterial infection (63)
**miR-146**	Linked to regulation of TLR signaling (66)
	Clearance of bacterial infection (63)
**miR-148**	Inhibition of demethylation Foxp3 (43, 67)
	Suppression of TGFβ signaling *via* SMAD (68)
	Regulation of DNMT1 and DNMT3, epigenetic homeostasis of DNA methylation (69)
**miR-155**	Anti-inflammatory effects (70)
	Regulation of TLR signaling (66)
	Induction of Tregs (71)
**miR-181**	Anti-inflammatory effects (72)
	NFkB signaling (73)
**miR-223**	Linked to infection and inflammation (74)
	Eosinophil function (75)

Milk-derived EVs have a particularly resilient lipid bilayer membrane, which serves to protect miRNAs from degradation caused by low pH and rich enzymatic environments, as seen in the gastro-intestinal tract. Minimal loss of RNA was observed after exposing milk EVs to digestive juices such as saliva and gastric, pancreatic, and bile juice (39). Also, there are some studies showing that miRNA from milk EVs can be found in blood and organs from humans and mice (41, 76). Additionally, using the *in vitro* TNO intestinal model-1, representing the gastro-intestinal tract from stomach to small intestine, it was shown that 2 h of ‘digestion’ resulted only in a minor loss of the abundant miR-223 and miR-125b (40). These findings indicate that mEVs can reach the small intestine without losing their integrity. Besides their resilience to low pH and enzymatic degradation, milk EVs can also withstand high temperatures, as milk EVs isolated from store-bought pasteurized milk are still bioactive (77). We will further discuss the bioactivity and effects of milk EVs on various cell types below.

## Immunomodulatory Properties of Extracellular Vesicles

Milk EVs, and EVs in general, have interesting immunomodulatory properties. Many studies have shown involvement of EVs in the regulation of the immune response, acting as both enhancers and dampeners of the immune system, depending on the source and type of vesicle and the receiving cell type. Immunosuppressive EVs are naturally present in the body, including T cell-derived EVs, which have been shown to downregulate antigen presentation by antigen-presenting cells (78). Additionally, stem cell-derived EVs are vastly investigated for their immune-modulatory properties [reviewed in (79)] Both embryonic stem cells (ESCs) and mesenchymal stem cells (MSCs) are producers of EVs with strong immunosuppressive capacities, similar to that found using stem cells as therapeutics themselves. Finally, research, including our own at the Radboudumc, has shown the anti-inflammatory effects of milk-derived EVs, using human breast milk as well as bovine colostrum and store-bought pasteurized milk. Although it is not completely elucidated which factors within the EVs contribute to these immunosuppressive capacities, a number of proteins and miRNAs are likely candidates.

Despite their immunosuppressive role, in many diseases EVs have been found to enhance inflammation as well (80). For example, EVs derived from synovial fluid of RA patients contain high levels of TNFα and have been shown to delay activated T cell-mediated cell death, possibly contributing to the pathogenesis in RA (81). Similarly, sarcoidosis patients have EVs in their bronchoalveolar fluid, which show pro-inflammatory properties (82). Macrophage-derived EVs can also carry alarmins and contribute to bone homeostasis (83). It is noteworthy that the membrane receptor composition, cellular metabolism, and role in the disease process of the recipient cell may also determine the net outcome of the EV response.

## T Cell Activation and Differentiation by Extracellular Vesicles

Activated CD4+ T cells are found in inflammatory infiltrates of the rheumatoid synovium (84), and the hallmark cytokine for Th17 cells, IL-17, is spontaneously produced in synovial explant cultures of RA donors (85). In experimental animal models for RA, such as collagen-induced arthritis and adjuvant arthritis, the disease can be transferred by autoreactive T cells (86). Collagen-induced arthritis is clearly attenuated in IL-17 deficient mice (87), and in IL1rn-deficient mice, spontaneous arthritis is completely prevented in the absence of IL-17 (88). Another important cytokine in the pathophysiology of RA and key in Th17 differentiation is IL-23, which is detectable in RA synovial joints (89, 90). In patients with RA, the Th17 and regulatory T cell (Treg) balance is skewed in favor of Th17 development, contributing to a break in tolerance and autoimmunity (91).

A strong candidate to modulate T cell function, especially Th17 and Treg cells, is transforming growth factor-beta (TGFβ). TGFβ has been found on the surface of EVs from a number of different origins, including mast cells (92), tumor cells (93, 94), but also milk-derived EVs (77) and intestinal epithelial cells (IECs) (95). Most notable is a study by Cai et al. who used TGF-β1 gene-modified dendritic cells (DCs) to produce immunosuppressive EVs, which were able to attenuate inflammatory bowel disease *in vivo*. A significant prevention of weight loss, decreased disease activity scores, as well as reduced intestinal bleeding was observed after the administration of TGF-β1-EVs (96).

Ogino et al. speculate the underlying mechanism could be *via* the induction of Tregs, which are known to downregulate Th17 cells and thereby suppress colonic inflammation (97). Interestingly, milk EVs from both human (98) and bovine milk (14) have been shown to promote Treg differentiation. Admyre et al. (98) were among the first to show Treg differentiation induced by EVs isolated from colostrum and mature breast milk. Their functional analyses showed that milk EVs can inhibit anti-CD3-induced IL-2 and IFN-γ production by T cells and simultaneously increase the number of Treg cells *in vitro.* A potential link to the prevention of asthma by Tregs suppressing Th2 responses was later suggested (99). Additionally, Zonneveld et al. have recently reported that human milk EVs can directly inhibit CD4+ T helper cell activation without inducing tolerance (100). In experimental arthritis studies, our research group at the Radboudumc found circumstantial evidence for this effect on T cells, as mice treated with bovine mEVs showed a marked reduction in Tbet (Th1) and RORyT (Th17) expression in splenocytes. Although no changes were observed in the Treg subset *in vivo*, we were able to confirm that EVs from pasteurized bovine milk enhanced Treg differentiation *in vitro.* Further research is needed to elucidate if the route of EV administration, as well as the timing in the developing immune response, determines the net outcome of the EVs, as has been demonstrated for therapeutic viral vectors and stem cells (101).

## Microbiome and Barrier Function in RA

Several studies in RA patients and animal models showed that dysbiosis of the gut microbiota induces an inflammatory response and is associated with disease progression of RA (102). For instance, new onset rheumatoid arthritis (NORA) patients have enriched levels of *Prevotella copri* in their gut, and this correlates with enhanced susceptibility to RA (8). Interestingly, germ-free mice inoculated with *P. copri*-dominated fecal samples from RA patients developed arthritis in a Th17-dependent manner (103). Of great interest, our group showed that these alterations in intestinal microbiome may precede the development of arthritis, as our study showed that the intestinal microbiome undergoes marked changes in the preclinical phase of collagen-induced arthritis (26). It is also known that the intestinal barrier is changed before the onset of RA. Ileal mucosal biopsies from treatment-naïve NORA patients and active RA patients showed a reduced expression of tight junction proteins claudin-1 and occludin compared to healthy controls on mRNA level and histology (23). Also, increased levels of CD3+ T cells, macrophages, and B cells were found in the lamina propria of NORA patients (23). Unfortunately, RA patients are often treated with methotrexate, but this DMARD is known to increase intestinal permeability (104, 105). Interestingly, patients with RA successfully treated with DMARDs show partial restoration of eubiotic gut microbiome, suggesting a crucial role of microbiota in treatment efficacy (106).

## Milk EVs Promote Gut Barrier Integrity

In RA, the gut–joint axis is in part related to the observation of leaky guts in some of these patients as cause of the elevated levels of bacterial cell wall fragments as well as bacterial DNA in the joints of these patients (107–111). The mucosal barrier is an important line of defense against invasion, infection, and bacterial dissemination. Underneath the epithelial cells lies the lamina propria, where T cells, macrophages, B cells, and plasma cells are present, and dendritic cells promote the differentiation of Th17 and Treg cells (112). The intestinal epithelial barrier prevents the entry of microbes into this lamina propria (112). Milk components have a protective effect on the intestine by improving its barrier function and microbiome diversity and limiting inflammatory processes. Milk EVs, from different species, show a similar tendency (113–115). Most milk EV studies focusing on barrier function study the functional effects on the epithelial cells, often using cell lines or animal models for necrotizing enterocolitis (NEC). Porcine milk EVs have been shown to promote cell proliferation of intestinal epithelial cells from newborn (unsuckled) piglets (IPEC-J2 cells), as well as, promote intestinal tract development *in vivo*, as shown by increased villus height, crypt depth, and higher expression of CDX2, PCNA, and IGF-1R (116). Similarly, milk EVs also promote epithelial cell growth, potentially *via* activation of the MAPK pathway (117). Additionally, milk EVs were able to protect mice from intestinal injuries caused by NEC (118). Reduced intestinal inflammation (myeloperoxidase expression) was observed, as well as an increase in goblet cell activity (MUC2+ and GRP94+ cells), highlighting the potential novel application of milk-derived EVs in the prevention of NEC development. Several studies using human milk EVs show comparable results. Martin et al. found that human breast milk-derived EVs had a protective effect on intestinal epithelial cells, reducing oxidative stress-induced cell apoptosis (induced by H_2_0_2_) (119). The factors from EVs that promote the intestinal barrier function have not been identified, but the expression of *e.g.* polymeric immunoglobulin receptor on EVs could be of importance. This receptor mediates the transcytosis of dimeric IgA and polymeric IgM through the intestinal epithelial layer and by this, protects against bacterial overgrowth and invasion causing leakage. Interestingly, two cow milk EV subsets [isolated by ultracentrifugation 35,000 g (P35K) or isolated at 100,000 g (P100K)] were administered orally by gavage to healthy and DSS (dextran sodium sulfate)-treated mice. P35K EVs and P100K EVs (to a lesser extent) improved several outcomes associated with DSS-induced colitis; they restored intestinal impermeability, replenished mucin secretion, and modulated the gut microbiota (13).

## Therapeutic Use of Milk EVs

The use of milk EVs, either as stand-alone drug, drug carrier, or functional dietary component, is often suggested in recent years. Several research groups have studied the tolerance and safety of milk-derived EVs in animal models, administered either intravenously or by oral gavage, and the consensus is that they are well tolerated with no significant changes or slightly induced cytokine levels systemically (48, 76). Due to its composition, milk-derived EVs are highly biocompatible and have enhanced stability and limited immunogenicity, which gives them many advantages over traditional synthetic delivery vehicles, such as liposomes, indicating that they might be well tolerated. Furthermore, it has been demonstrated that milk-derived EVs are taken up in the gastro-intestinal tract after oral delivery *via* the neonatal Fc receptor, and they stay intact after absorption (120). This receptor mediates bidirectional transcytosis of IgG in epithelial cells and rescues albumin from intracellular degradation, thereby increasing plasma half-lives of these proteins.

As previously mentioned, milk-derived EVs have two important characteristics that make them very suitable as drug carriers; first of all, their lipid bilayer functions as a protective shell for drugs inside, and second, the efficient uptake of EVs results in improved bioavailability (**Box 4**) of the drug. Among one of the first studies is a large study undertaken by the group of Gupta, who developed a scalable isolation method for bulk production of milk-derived EVs that can act as carriers for chemotherapeutic agents (76). They used a number of different chemotherapeutics and chemoprotective compounds, including withaferin A, to test loading efficiency which varied between 10 and 40% depending on the agent. After confirming tumor growth inhibition by drug-loaded EVs *in vitro*, they compared efficacy of drug-loaded EVs to free drug in a long tumor xenograft model *in vivo* and found a significantly greater tumor inhibitory effect with drug-loaded EVs (76). A follow-up study, this time using paclitaxel-loaded EVs, demonstrated oral delivery also resulted in significant tumor growth inhibition in a tumor xenograft model (124). Additionally, the study confirmed the stability of paclitaxel-loaded EVs for storage up to four weeks at −80°C (124). Milk-derived vesicles have also been used as a novel delivery system for small interfering RNA (siRNA) in a therapeutic application against cancer (125, 126). Furthermore, when encapsulated in milk EVs, curcumin showed increased stability, solubility, and bioavailability (127). Of note, as discussed in the previous paragraphs, milk-derived EVs themselves already have a substantial immunoregulatory function, and even without loading, these vesicles can act as therapeutics. Additionally, the characterization of EVs to monitor potential differences is very important, and this is still a field of ongoing research.

Box 4 **Bioavailability and safety of milk EVs.**

Research has shown that milk EVs are easily taken up by several different cell types. Intestinal cells are particularly quick to take up milk EVs when exposed. Wolf et al. (121) showed that both Caco-2 and IEC-6, intestinal cell lines, are able to take up milk EVs as fast as within 15 min. Intestinal uptake of EVs is likely *via* receptor-mediated endocytosis by intestinal epithelial cells (transcellular transport) or paracellular transport *via* tight junctions. Interestingly, not all cells can take up milk EVs; for example undifferentiated THP-1 cells (monocytes) do not show uptake, whereas their differentiated counterpart (macrophages) do take up EVs (122), indicating there is a cell type or cell differentiation state specific mechanism at work. Besides *in vitro* uptake, several animal studies have shown uptake and biodistribution of milk EVs in mice (48, 123). Both oral intake and intravenous injection (i.v.) resulted in peak uptake in the liver and spleen of mice, after 24 and 3 h, respectively. Interestingly, miRNAs transfected into the milk EVs were found in several organs 6 and 12 h after oral gavage (123), confirming uptake *in vivo*. In the intestine, EVs could exert other additional effects due to their ability to spread, cross the mucus layer, and directly migrate to other tissues and/or interact with different cells of the immune system of the host. In healthy animals, the biocompatibility and safety have been tested, and extensive analysis confirmed that there were no systemic changes upon i.v. injection of milk EVs into mice (48). Blood levels of markers for liver damage (aspartate transaminase, alanine transaminase, and total bilirubin), kidney damage (blood urea nitrogen and creatinine) and hematological parameters were all unchanged (48).

## Future Research

There is increasing awareness that the gut plays a vital role in our overall health. The gut represents the largest surface area being exposed to our environment and is also the largest immune organ in our body. An enhanced intestinal leakiness, dysbiosis of the gut microbiome, and bowel inflammation are not only associated with diseases of the gut such as colitis and Crohn’s disease, but are also characteristic of many other systemic inflammatory diseases such as lupus, multiple sclerosis, psoriatic arthritis, systemic sclerosis, and RA (128–131). Strategies to target the gut, and especially its microbiome, using pro- and prebiotics (27) are under investigation and hold a promise as a therapeutic intervention for these diseases.

**Figure 1:**
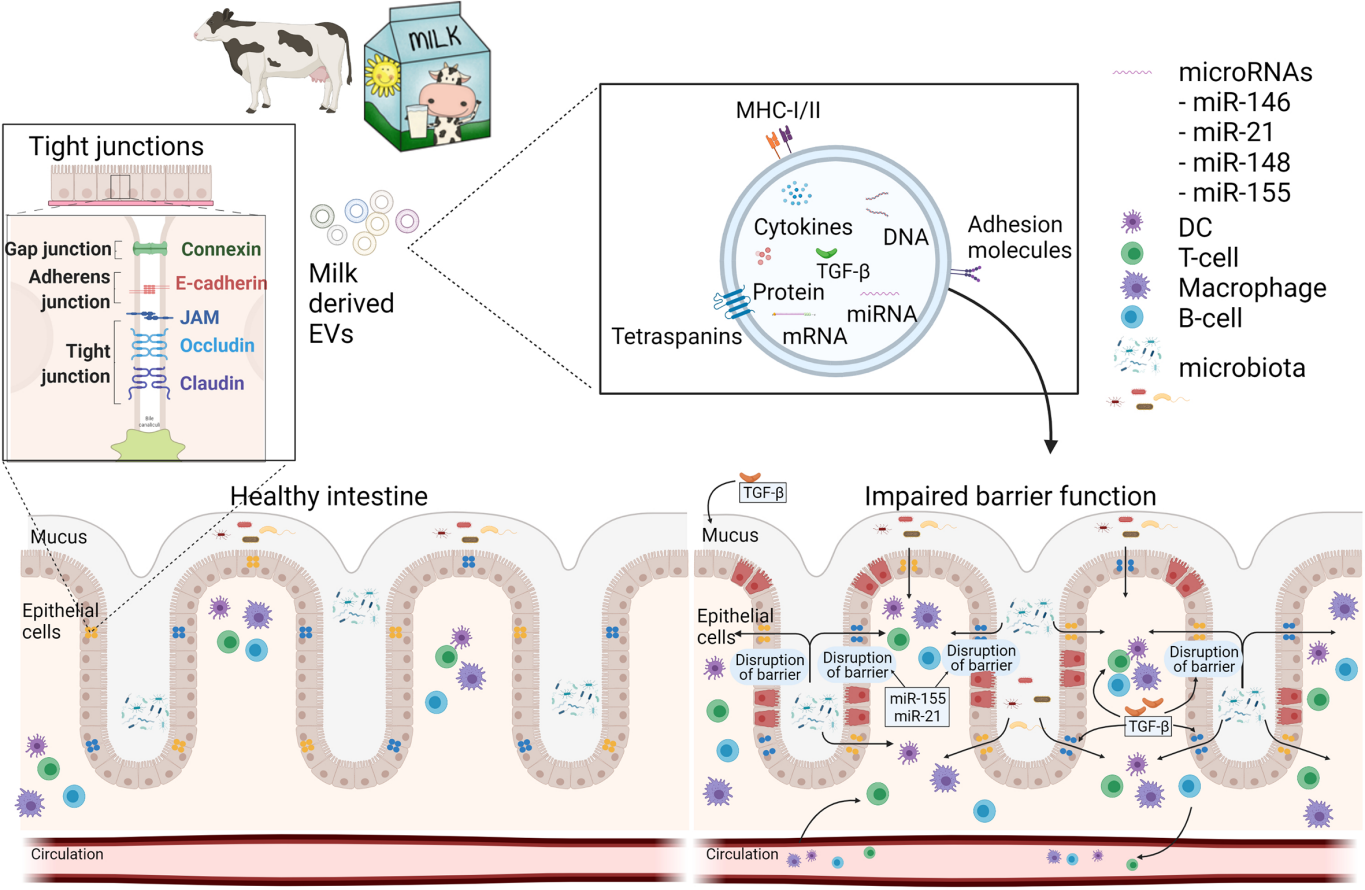
Schematic overview how milk EVs can modulate intestinal barrier function and immunity. A healthy intestine has an intact barrier of various intestinal cells and mucus. Milk-derived EVs may contribute to the restoration of an impaired barrier function during disease by increasing mucus production and expression of tight junctions *via* miRNAs and TGF-β. Furthermore, mEVs can act on immune cells, locally in the gut, or systemically *via* the circulation. This figure was in part created with BioRender and was licenced for use in publication (created with BioRender.com).

We hypothesize that milk-derived EVs could be a potential therapeutic strategy (**Figure 1**) in modulating the gut–joint axis in RA. Since the net effect of the total dairy matrix on human health is dependent on the health status of the individual, the product type of dairy, and individual preferences towards dairy products, several aspects need to be considered before such application could be implemented. The isolation of pure extracellular vesicles without other milk constituents like fat globules, milk proteins, lactose, and feed-derived milk components, would provide a widely applicable format of milk-derived EVs for therapeutic application. Pure mEVs would be preferred over more complete milk products, since lactose intolerance is prevalent in a large part of the world, and RA patients for example can have increased antibodies against food antigens including milk proteins of cows (132). The isolation procedure is important and should conform GMP guidelines.

One of the important aspects to tackle is the reproducibility of the efficacy of the milk EV product used. Another challenge is the translation of studies performed *in vitro* or in animals into humans.

More research is required to figure out what the active components of the milk-derived EVs are. Whether these are miRNAs, growth-factors, or other proteins, or a combination of these factors is important to understand. Whether further separation, based on size or content, into subpopulations of the heterogeneous population of EVs is required, needs attention. Another parameter determining the content of milk-EVs is the origin of milk, *i.e.* species (cow, camel, horse, goat or sheep), changes during lactation period, food intake, seasonal effects, and animal breeds used.

Additionally, we need to know if these vesicles are actively taken up *via* oral intake in humans and show similar effects to the mouse and *in vitro* models described here. Finally, standard practices for the isolation, especially on a larger scale, are required.

Over the years, many different isolation protocols have been developed for the isolation of milk-derived EVs. Each isolation protocol comes with its own strengths and pitfalls, which are nicely compared in a recent article by Maburutse et al. (133) Ultracentrifugation is the most used isolation method, either as a stand-alone procedure or in combination with further purification using density gradients, isoelectric precipitation, or size-exclusion chromatography (SEC). Several methods to lose the casein and whey proteins, followed by purification of the milk EV *via* ultracentrifugation, size exclusion chromatography, membrane affinity columns, or solid phase extraction have been reviewed (134–136). Which process is most applicable for upscaling, with the preservation of biological functionality of the milk EVs, needs to be validated.

Upfront milk testing and quality control will be an essential component in the milk processing and downstream EV isolation. Furthermore, the milk EV isolation methods that are used can influence the composition of the EV sample. As described by Provost, different subsets of milk EVs are present in commercial milk (137). They found that a milk EV subset, which pellets at low ultracentrifugation speeds, contains and protects the bulk of milk microRNAs from degradation. In addition, sample collection methods as well as storage conditions influence the quality of the EVs. Zonneveld et al. have shown that prolonged storage at 4°C and −80°C can lead to cell death which results in contamination of the EV population in human breast milk. Interestingly, the cow breed and even the diet of the cow can also influence the milk EV composition (137, 138). These are all important considerations in moving forward to establish a standardized, large-scale isolation protocol for milk EVs, ready to be used as potential therapeutics.

## Future Perspective and Final Consideration

Altogether, this review highlights the therapeutic potential of milk EVs to treat arthritis and inflammatory gut diseases. Once a suitable large-scale isolation method is established and it is confirmed that the vesicles retained their therapeutic potential *in vitro* and in mouse models of disease, we propose testing the bioavailability and safety in both human organoids (**Box 5**) and humans. It will not replace the current standards of care (DMARDs, biologicals) but will be a sophisticated supportive treatment by disrupting the pathogenic gut–joint axis.

Box 5 **Organoids.**

In the human body, the intestinal epithelial layer is exposed to the microbiome. Although the microbiome is separated from the enterocytes by a mucus layer, bacterial-derived metabolites can penetrate this mucus layer and affect growth, differentiation, and intestinal health. To mimic these interactions *in vitro*, there are several factors to take into consideration. For instance, the intestine consists of different cell types such as, stem, Paneth, goblet, enteroendocrine cells, and enterocytes. The recent development of intestinal organoid cultures in 3D and 2D, however, allows the use of more sophisticated cultures with all cell types present.

A second hurdle to take, and maybe the most difficult one, is the difference in growth (conditions) between human cells and bacteria. When bacteria are co-cultured with human cells, they will rapidly overgrow the culture and kill the human intestinal cells within hours. In addition, human intestinal cells require high oxygen levels, whereas most intestinal bacteria grow anaerobic. One way to solve these problems is by micro-injecting bacteria into the lumen of organoids/spheroids (139). Williamson et al. injected human fecal microbiota and showed that even oxygen-sensitive anaerobic taxa are maintained for at least 96 h. However, when longer studies are required, the group of Donald E. Ingber has developed an anaerobic human intestine and microbiome-on-a-chip system (140). Although they used Caco2 cells and endothelial cells instead of organoids, they nicely demonstrated that it is possible to create an oxygen gradient that allows the growth of human intestinal cells combined with anaerobic bacteria. The next step would be to apply 2D grown human intestinal organoids, replacing Caco2 cells in this system.

## Author Contributions

JA and BP wrote the first draft of the manuscript and share first authorship. AF, AB, and JK wrote a part of the text boxes. All authors contributed to the article and approved the submitted version.

## Funding

This study was powered by Health~Holland, Top Sector Life Sciences & Health (grant LSHM19108 SGF) and the grant was allowed to FL.

## Conflict of Interest

Authors ALF and RJJN was employed by company FrieslandCampina.

The remaining authors declare that the research was conducted in the absence of any commercial or financial relationships that could be construed as a potential conflict of interest.

## Publisher’s Note

All claims expressed in this article are solely those of the authors and do not necessarily represent those of their affiliated organizations, or those of the publisher, the editors and the reviewers. Any product that may be evaluated in this article, or claim that may be made by its manufacturer, is not guaranteed or endorsed by the publisher.

## Abbreviations

EV, extracellular vesicle; RA, rheumatoid arthritis, GF, germ-free; TLR, toll-like receptor; MSC, mesenchymal stem cell; IEC, intestinal epithelial cell; NEC, necrotizing enterocolitis; SEC, size-exclusion chromatography; MVE, multivesicular endosome.
